# The contributions of deleterious rare alleles in *NLRP12* and inflammasome-related genes to polymyalgia rheumatica

**DOI:** 10.1038/s41598-024-51320-3

**Published:** 2024-01-04

**Authors:** Takashi Higuchi, Shomi Oka, Hiroshi Furukawa, Shigeto Tohma

**Affiliations:** Department of Rheumatology, NHO Tokyo National Hospital, 3-1-1 Takeoka, Kiyose, 204-8585 Japan

**Keywords:** Genetics, Immunology, Rheumatology

## Abstract

Polymyalgia rheumatica (PMR) is a chronic inflammatory disease characterized by arthralgia and myalgia of the shoulder and hip girdles, and fever. PMR is linked to autoimmune diseases and autoinflammatory disorders. Exome sequencing has revealed the roles of rare variants in some diseases. Causative genes for monogenic autoinflammatory disorders might be candidate genes for the selective exome analysis of PMR. We investigated rare variants in the coding and boundary regions of candidate genes for PMR. Exome sequencing was performed to analyze deleterious rare variants in candidate genes, and the frequencies of the deleterious rare alleles in PMR were compared with those of Japanese population controls. Deleterious rare alleles in the *NLRL12* gene were associated with PMR (*P* = 0.0069, *P*c = 0.0415, odds ratio [OR] 4.49, 95% confidence interval [CI] 1.79–11.27). A multigene analysis demonstrated the deleterious rare allele frequency of the candidate genes for autoinflammatory disorders was also increased in PMR (*P* = 0.0016, OR 3.69, 95%CI 1.81–7.54). The deleterious rare allele frequencies of the candidate genes including *NLRP12* were increased in PMR patients, showing links to autoinflammatory disorders in the pathogenesis of PMR.

## Introduction

Polymyalgia rheumatica (PMR) is a chronic inflammatory disease characterized by arthralgia and myalgia of shoulder and hip girdles, morning stiffness, and fever^1^. PMR affects people over 50 years of age and is frequently complicated with giant cell arteritis in European populations^2^, but not in Japanese populations (0–1%)^3,4^. Several studies reported a potential association between PMR and malignancy, although this is still controversial^5–8^. Acute phase reactants are increased in PMR and corticosteroid treatment is effective for PMR. It is sometimes difficult to discriminate PMR from older age onset rheumatoid arthritis (RA). Of note, no specific autoantibodies have been detected in PMR patients and the pathogenesis of PMR is still unknown, although it is likely to be influenced by genetic and environmental factors.

Previous studies demonstrated the genetic associations of *HLA-DRB1, ICAM1, CCL5, TNF, IL1RN, IL6,* and *NOS3* genes with PMR using a candidate gene approach^2,9–16^, however, no genome-wide association studies have been reported for PMR. On the other hand, many genetic studies, including genome-wide association studies, have been performed in giant cell arteritis^17–20^. Because PMR is frequently associated with giant cell arteritis in European, the overlap of susceptibility genes is possible. Susceptibility genes for PMR detected in Japanese could be easily discriminated from those for giant cell arteritis, because of the low overlap rates. Thus, genetic studies of Japanese PMR are necessary to understand the specific pathogenesis of PMR.

It is currently thought that PMR is linked to autoimmune diseases and autoinflammatory disorders^2,21^. Disease onset of autoinflammatory disorders and PMR is acute, though that of autoimmune diseases is subacute. Rapid remission is achieved by the treatment in autoinflammatory disorders and PMR, but not in autoimmune diseases. Acute phase reactants are remarkably increased in autoinflammatory disorders and PMR; these are not increased in autoimmune diseases. No specific autoantibodies are detected in autoinflammatory disorders and PMR. However, autoantibodies are frequently detected in autoimmune diseases. Thus, PMR is linked to autoinflammatory disorders.

Exome sequencing analyses were employed to reveal the roles of deleterious rare variants with higher effect sizes in some polygenic diseases^22–27^, since exome sequencing with enough depth is reliable. Deleterious rare variants include nonsense variants, frameshift variants, splice site variants, and deleterious missense variants. These deleterious rare variants could affect functions of proteins coded by the genes. Nonsense variants, frameshift variants, and splice site variants are loss of function variants, but deleterious missense variants might be gain of function variants. Thus, exome sequencing analyses on deleterious rare variants focused on the roles of rare functional variants with higher effect sizes to reveal causative genes in polygenic diseases.

Causative genes for monogenic autoinflammatory disorders have been reported. Monogenic autoinflammatory disorders with defects in inflammasome-related genes include familial Mediterranean fever, hyper IgD syndrome, and familial cold autoinflammatory syndromes, which are characterized by fever, arthralgia, myalgia, and urticaria^28^. The causative genes for these autoinflammatory disorders include *NLRP12, PLCG2, NLRP3, MEFV, NLRC4,* and *MVK*. Deleterious rare variants in these genes were detected in undiagnosed patients with fever, arthralgia, or myalgia^29–31^. However, few studies of deleterious rare alleles as the causative genes for autoinflammatory disorders in patients with PMR have been conducted. Of note, PMR also has links to autoinflammatory disorders, and thus, causative inflammasome-related genes for autoinflammatory disorders are good candidates for selective exome analysis to reveal the genetic predisposition to PMR. Here, we investigated rare variants in the coding and boundary regions of the candidate genes in PMR patients and compared the frequencies of deleterious rare alleles in these patients with those in Japanese population controls.

## Materials and methods

### Patients and controls

Twenty-eight patients with PMR were recruited at the Tokyo National Hospital. These PMR patients were native Japanese living in Japan and were not related to each other. The patients fulfilled the 2012 Provisional Classification Criteria for Polymyalgia Rheumatica^32^. Corticosteroid resistance was defined as the reappearance of clinical symptoms associated with the elevation of acute-phase reactants during the tapering of corticosteroids. These patients were treated with disease modifying anti-rheumatic drugs and corticosteroids for the relapse of PMR. Allele frequencies in candidate genes in Japanese populations were obtained with reference to the 38KJPN panel from the Tohoku Medical Megabank Organization (n = 38,722, https://jmorp.megabank.tohoku.ac.jp/, accessed on 28 April 2023)^33^.

This study was reviewed and approved by the Research Ethics Committees of Tokyo National Hospital. Written informed consent was obtained from all the participants. This study was conducted in accordance with the principles expressed in the Declaration of Helsinki.

### Exome sequencing followed by selective candidate gene analyses

Genomic DNA was extracted from peripheral blood of the PMR patients by the phenol chloroform extraction method and quantitated by Qubit Fluorometer (Thermo Fisher Scientific Inc., Waltham, MA). Exome sequence libraries were constructed from the genomic DNA using Acoustic Solubilizer Covaris (Covaris, Woburn, MA), Twist Library Preparation Kit Mechanical Fragmentation (Twist Bioscience, South San Francisco, CA), and Twist Comprehensive Exome Panel (Twist Bioscience). The exome sequence libraries were qualified by Agilent 2100 BioAnalyzer (Agilent Technologies, Santa Clara, CA) and sequenced on a NovaSeq 6000 (Illumina, San Diego, CA). Sequence reads were mapped to the *Homo sapiens* genome assembly of GRCh38 and small variant calling was conducted by DRAGEN Bio-IT Platform (Illumina) under the condition of default small variant hard filtering (https://jp.support.illumina.com/content/dam/illumina-support/help/Illumina_DRAGEN_Bio_IT_Platform_v3_7_1000000141465/Content/SW/Informatics/Dragen/GPipelineVarCalFilt_fDG.htm).

Because PMR share several symptoms, arthralgia, myalgia, and fever, with monogenic autoinflammatory disorders with defects in inflammasome-related genes, the causative genes for these autoinflammatory disorders were good candidates. A total of six genes, *NLRP12, PLCG2, NLRP3, MEFV, NLRC4,* and *MVK,* were on a list of inflammasome-related genes^28^ and candidates in the present study. Variants in the coding regions and boundary regions of the candidate genes were analyzed. Variants with minor allele frequencies less than 1% in the 38KJPN panel were included^27^ and synonymous variants and intronic variants outside the splice sites (two bases) were excluded. Deleterious missense variants (probably damaging or possibly damaging in PolyPhen-2 HumDiv or HumVar) were defined by PolyPhen-2 (http://genetics.bwh.harvard.edu/pph2/index.shtml)^34^. An allele number of deleterious rare variants (deleterious missense variants, nonsense variants, frameshift variants, and splice site variants) in each candidate gene was compared between the PMR patients and Japanese population controls. A total number of deleterious rare alleles in the inflammasome-related genes tested were used for the burden test of multigene analysis to elucidate whether the pathways of inflammasome-related genes are involved in the pathogenesis of PMR: the total number of deleterious rare variants in the six candidate genes in the PMR populations were compared with that in Japanese populations^25^.

### Statistical analysis

The number of deleterious rare alleles in each candidate gene or the total number in the six candidate genes in PMR patients were compared with those in Japanese population controls by Fisher's exact test using 2 × 2 contingency tables under the allele model^22,23^. The clinical characteristics of the PMR patients with deleterious rare alleles were compared with those without by the Mann–Whitney *U*-Test or Fisher's exact test using 2 × 2 contingency tables. The corrected *P* (*P*c) value was calculated to correct for multiple testing by the Bonferroni correction method. Statistical significance was defined as *P*c < 0.05.

### Ethics approval and consent to participate

This study was reviewed and approved by the Research Ethics Committees of Tokyo National Hospital. Written informed consent was obtained from all the participants. This study was conducted in accordance with the principles expressed in the Declaration of Helsinki.

## Results

### Demographics of PMR patients

The demographics of PMR patients are shown in Table 1. Mean (standard deviation [SD]) age of the PMR patients was 76.2 (6.2) years. Of 28 PMR patients, 17 (60.7%) were male. Mean (SD) of age at onset was 75.9 (6.5) years. Complication of malignancy was observed in 9 (32.1%) patients. No PMR patient was complicated with giant cell arteritis. Corticosteroid resistance was observed in 11 (39.3%) patients. Autoantibodies were detected in a small subpopulations of PMR patients. Acute phase reactants were markedly increased in the PMR patients. The incidence rate and the prevalence of PMR in Japan were estimated from the incidence ratio and the prevalence ratio between RA and PMR in Tokyo National Hospital. The incidence rate of RA was reported to be 22 per 100,000 people in Japan^35^. Since the incidence ratio between RA and PMR was 0.67 in Tokyo National Hospital from 2019 to 2022 (RA: 11.0, PMR: 7.3), PMR was estimated to occur in 18,000 peoples in every year in Japan using the demographic statistics of Japan in 2022 (https://www.stat.go.jp/data/jinsui/2022np/zuhyou/05k2022-1.xlsx). The incidence rate of PMR was also estimated to be 30 per 100,000 people aged 50 years and older in Japanese populations. The prevalence of RA was reported to be 750 per 100,000 people aged 16 years and older in Japanese populations^36^. Since the prevalence ratio between RA and PMR was 0.20 in Tokyo National Hospital in 2022 (RA: 148, PMR: 29), a total of 160,000 PMR patients were estimated to be suffering from PMR in Japan using the demographic statistics of Japan in 2022. The prevalence of PMR was estimated to be 260 per 100,000 people aged 50 years and older in Japanese populations.

**Table 1:** Demographics of PMR patients.

Number	28
Mean age, years (SD)	76.2 (6.2)
Male, n (%)	17 (60.7)
Age at onset, years (SD)	75.9 (6.5)
Complication of malignancy, n (%)	9 (32.1)
Complication of giant cell arteritis, n (%)	0 (0.0)
Corticosteroid resistance, n (%)	11 (39.3)
RF positive (> 15[U/ml]), n (%)	2 (7.1)
ACPA positive (> 4.5[U/ml]), n (%)	2 (7.1)
ANA positive (> 40X), n (%)	2 (7.4)
ESR (mm/h)	80.1 (27.2)
CRP (mg/dl)	8.7 (6.1)
MMP3 (ng/ml)	211.1 (126.0)

### Associations of deleterious rare alleles with PMR

Exome sequencing was performed with an average alignment coverage over the target region of 256.0 and at least tenfold alignment coverage was obtained for 97.6% of the target sequences. Nine deleterious rare alleles were detected in the candidate genes for autoinflammatory disorders in PMR patients and these variants included eight missense deleterious variants (Supplementary Table S1). These alleles included five in *NLRP12* (chr19:53794089, rs146786265, c.3149C > T, p.Ala1050Val, 2 alleles; chr19:53804094, rs1435753276, c.2446G > T, p.Ala816Ser, 1 allele; chr19:53811030, rs377594629, c.629C > T, p.Pro210Leu, 1 allele; chr19:53824150, rs762604819, c.25G > A, p.Gly9Ser, 1 allele), two in *PLCG2* (chr16:81895901, c.1167C > G, p.Ile389Met, 1 allele; chr16:81912602, rs751244429, c.1940A > C, p.Tyr647Ser, 1 allele), one in *NLRP3* (chr1:247434110, rs772009059, c.2335C > T, p.Arg779Cys, 1 allele), and one in *MEFV* (chr16:3243343, rs55901263, c.2144C > G, p.Pro715Arg, 1 allele). No homozygous or compound heterozygous patient was detected, one patient possessed rs1435753276 in *NLRP12* and rs751244429 in *PLCG2,* and no other patients possess more than one deleterious variants. Deleterious rare allele frequencies in the candidate genes for autoinflammatory disorders in PMR patients and the Japanese population controls are shown in Table 2. The deleterious rare allele frequency of *NLRP12* was significantly increased in PMR patients compared with controls (*P* = 0.0069, *P*c = 0.0415, odds ratio [OR] 4.49, 95% confidence interval [CI] 1.79–11.27). In the multigene analysis, the deleterious rare allele frequency of the candidate genes for autoinflammatory disorders was also increased in PMR patients compared with controls (*P* = 0.0016, OR 3.69, 95%CI 1.81–7.54). Thus, the deleterious rare allele frequencies of the candidate genes including *NLRP12* were increased in PMR patients.

**Table 2:** Burden of deleterious rare alleles in PMR patients and controls.

	Case (2n = 56)	Control (2n = 77,444)	*P*	OR	95%CI	*P*c
*NLRP12*	5 (8.9)	1654 (2.1)	0.0069	4.49	(1.79–11.27)	0.0415
*PLCG2*	2 (3.6)	474 (0.6)	0.0466	6.01	(1.46–24.74)	0.2798
*NLRP3*	1 (1.8)	755 (1.0)	0.4226	1.85	(0.26–13.36)	NS
*MEFV*	1 (1.8)	430 (0.6)	0.2683	3.26	(0.45–23.59)	NS
*NLRC4*	0 (0.0)	451 (0.6)	1.0000	1.51	(0.09–24.46)	NS
*MVK*	0 (0.0)	56 (0.1)	1.0000	12.12	(0.74–198.59)	NS
Multigene analysis	9 (16.1)	3820 (4.9)	0.0016	3.69	(1.81–7.54)	NA

Allele frequencies are shown in parentheses (%). Deleterious rare allele frequencies of PMR patients were compared with those of Japanese population controls by Fisher's exact test using 2 × 2 contingency tables under the allele model. The corrected *P* (*P*c) value was calculated for the correction of multiple testing by the Bonferroni method.

*PMR* polymyalgia rheumatica, *OR* odds ratio, *CI* confidence interval, *NS* not significant, *NA* not applicable.

### Demographic features of PMR patients with or without deleterious rare alleles in the candidate genes

The clinical features of PMR patients with or without deleterious rare alleles in the candidate genes for autoinflammatory disorders were compared (Table 3). The complication rate of malignancy in PMR patients with deleterious rare alleles in the candidate genes tended to be lower than in those without (*P* = 0.2144).

**Table 3:** Comparison of the demographics between PMR patients with or without deleterious rare alleles.

Phenotype	Deleterious rare allele (+) (n = 8)	Deleterious rare allele (−) (n = 20)	*P-*value
Mean age, years (SD)	76.6 (5.1)	76.1 (6.7)	0.6460
Male, n (%)	5 (62.5)	12 (60.0)	*1.0000
Age at onset, years (SD)	76.1 (5.6)	75.8 (7.0)	0.6102
Complication of malignancy, n (%)	1 (12.5)	8 (40.0)	*0.2144
Complication of giant cell arteritis, n (%)	0 (0.0)	0 (0.0)	*1.0000
Corticosteroid resistance, n (%)	4 (50.0)	7 (35.0)	*0.6715
RF positive (> 15[U/ml]), n (%)	0 (0.0)	2 (10.0)	*1.0000
ACPA positive (> 4.5[U/ml]), n (%)	0 (0.0)	2 (10.0)	*1.0000
ANA positive (> 40X), n (%)	0 (0.0)	2 (10.5)	*1.0000
ESR (mm/h)	78.8 (22.0)	80.7 (29.9)	0.7388
CRP (mg/dl)	9.5 (7.7)	8.3 (5.5)	0.6472
MMP3 (ng/ml)	190.3 (108.9)	220.3 (134.8)	0.5976

Average values or numbers are shown. Standard deviations or percentages are shown in parentheses. Associations were analyzed between PMR patients with or without deleterious rare alleles by the Mann–Whitney *U*-test or Fisher's exact test using 2 × 2 contingency tables.

*PMR* polymyalgia rheumatica, *RF* rheumatoid factor, *ACPA* anti-citrullinated peptide antibody, *ANA* antinuclear antibody, *ESR* erythrocyte sedimentation rate, *CRP* C-reactive protein, *MMP3* matrix metalloproteinase 3.

*Fisher's exact test was used.

### Allele frequencies of the MEFV gene in PMR patients and controls

The *MEFV* gene exceptionally includes variants with minor allele frequencies ≥ 1%, which are responsible for the pathogenesis of familial Mediterranean fever^37^. Those responsible variants with minor allele frequencies ≥ 1% in the *MEFV* gene were selected for additional analyses. The allele frequencies of these variants were compared between PMR patients and the Japanese population controls (Supplementary Table S2). No variants were associated with PMR. These data suggested that variants with minor allele frequencies ≥ 1% in *MEFV* were not responsible for the pathogenesis of PMR.

## Discussion

The present study revealed an association between deleterious rare alleles of *NLRP12* and PMR. The *NLRP12* gene encodes a nucleotide-binding oligomerization domain-like receptor with a pyrin domain and the NLRP12 protein inhibits the activation of NF-κB and forms the NLRP12 inflammasome. Mutations of the *NLRP12* gene cause familial cold autoinflammatory syndrome 2, which is characterized by rash, fever, arthritis, conjunctivitis, and leukocytosis after cold exposure^28^. These patients are treated with canakinumab (anti-interleukin-1β antibody) or anakinra (interleukin-1 receptor antagonist). In the multigene analysis, deleterious rare alleles of the causative genes for monogenic autoinflammatory disorders were associated with PMR. This panel includes *PLCG2*, *NLRP3*, and *MEFV.* The *PLCG2* gene encodes phospholipase Cγ2 and the *NLRP3* gene encodes a cryopyrin; these proteins are related to the NLRP3 inflammasome. Mutations in the *PLCG2* and *NLRP3* genes cause familial cold autoinflammatory syndrome 3 and 1, respectively. The *MEFV* gene encodes a pyrin that forms the pyrin inflammasome. Mutations of the *MEFV* gene cause familial Mediterranean fever characterized by recurrent episodes of fever, peritonitis, and arthritis. These patients are also treated with canakinumab or anakinra. Few association studies of these genes with PMR have been reported to date. The results of the current study indicate the potential links to autoinflammatory disorders in the pathogenesis of PMR. Because autoantibodies against the ferritin heavy chain in PMR were reported^38,39^, PMR has some characteristics of autoimmune diseases. Our results also suggested a potential treatment for some PMR subsets linked to autoinflammatory disorders might be canakinumab or anakinra. Important roles of interleukin (IL)-1β are found in autoinflammatory disorders, but roles of IL-6 are highlighted in autoimmune diseases and PMR^21^. Associations of *HLA* were reported in autoimmune diseases and PMR; *HLA* is not associated with autoinflammatory disorders^2^. Thus, the molecular pathophysiology of PMR was linked to that of autoimmune diseases and two aspects of PMR were suggested.

The complication rate of malignancy in PMR patients with deleterious rare alleles in the candidate genes tended to be lower than in those without. These data suggested the heterogeneity of PMR patients: one PMR subset with deleterious rare alleles in the candidate genes and another with malignancy.

The associations of common variants in *HLA-DRB1*, *ICAM1*, *CCL5*, *TNF*, *IL1RN*, *IL6*, and *NOS3* with PMR have been reported^2,9–16^. *HLA-DRB1* is one of the strongest genetic factors for the predisposition to autoimmune diseases; PMR, giant cell arteritis, and RA have similar patterns of *HLA* association^2,40^. The roles of the other common variants in the pathogenesis of PMR should be elucidated by future genome-wide association studies, since PMR is considered to be a polygenic disease. No variants with minor allele frequencies ≥ 1% in the *MEFV* gene were associated with PMR. Although variants with minor allele frequencies ≥ 1% are responsible for the pathogenesis of familial Mediterranean fever, these variants were not responsible for the pathogenesis of PMR.

To the best of our knowledge, this is the first study of the association of deleterious rare alleles in *NLRP12* with PMR. Additionally, the frequencies of deleterious rare alleles in inflammasome-related genes were also increased in PMR. This study revealed the genetic links to autoinflammatory disorders in the pathogenesis of PMR. Since PMR is not frequently complicated with giant cell arteritis in Japan, the susceptibility genes for PMR detected in this study was discriminated from those for giant cell arteritis. There were some limitations in the present study. Because of the low frequencies of deleterious variants, the small number of candidate inflammasome-related genes, and the modest sample size, nine deleterious rare alleles were detected in the PMR patients of this study. As a result, the association of the deleterious rare alleles was limited. Furthermore, this was a single-center study in Japan. The results of our study should be confirmed in future larger scale multi-center and multi-ethnic studies to confirm the roles of the pathways of inflammasome-related genes in the pathogenesis of PMR. In future, precision medicine of some subsets of PMR could be established using canakinumab or anakinra.

### Supplementary Information

Supplementary Table S1.

Supplementary Table S2.

## Data Availability

Data supporting the findings of this study are presented in the paper and the supplementary file. Other data are available from the authors upon reasonable request. However, the clinical information and genotype data of each participant are not available under the conditions of informed consent mandated by the Act of the Protection of Personal Information.
