# Supplementary material for: The contributions of deleterious rare alleles in NLRP12 and inflammasome-related genes to polymyalgia rheumatica
Source: Sci Rep. 2024 Jan 4;14:490. doi: 10.1038/s41598-024-51320-3 (PMC10767114; doi:10.1038/s41598-024-51320-3)
Supplement: Supplementary file 1 — Supplementary Table S1. [file 41598_2024_51320_MOESM1_ESM.pdf]

Supplementary Table S1. Deleterious rare variants in the PMR patients.

Gene	Location	Reference ID number	Nucleic acid change	Amino acid change	Number of deleterous alleles in the PMR patients	Frequencies in the Japanese populations
NLRP12	chr19:53794089	rs146786265	c.3149C>T	p.Ala1050Val	2 alleles	0.008057
	chr19:53804094	rs1435753276	c.2446G>T	p.Ala816Ser	1 allele	0.000026
	chr19:53811030	rs377594629	c.629C>T	p.Pro210Leu	1 allele	0.001304
	chr19:53824150	rs762604819	c.25G>A	p.Gly9Ser	1 allele	0.001588
PLCG2	chr16:81895901		c.1167C>G	p.Ile389Met	1 allele	0.000013
	chr16:81912602	rs751244429	c.1940A>C	p.Tyr647Ser	1 allele	0.000956
NLRP3	chr1:247434110	rs772009059	c.2335C>T	p.Arg779Cys	1 allele	0.000310
MEFV	chr16:3243343	rs55901263	c.2144C>G	p.Pro715Arg	1 allele	0.000077

PMR: polymyalgia rheumatica.
