# Supplementary material for: The contributions of deleterious rare alleles in NLRP12 and inflammasome-related genes to polymyalgia rheumatica
Source: Sci Rep. 2024 Jan 4;14:490. doi: 10.1038/s41598-024-51320-3 (PMC10767114; doi:10.1038/s41598-024-51320-3)
Supplement: Supplementary file 2 — Supplementary Table S2. [file 41598_2024_51320_MOESM2_ESM.pdf]

Supplementary Table S2. Allele frequency of *MEFV* in the PMR patients and controls.

MEFV allele	Case (2n=56)	Control (2n=77444)	*P*-value	OR	95%CI
S503C	0 (0.0)	1950 (2.5)	0.4067	0.34	(0.02-5.55)
R408Q	2 (3.6)	3671 (4.7)	1.0000	0.74	(0.18-3.05)
P369S	3 (5.4)	4239 (5.5)	1.0000	0.98	(0.31-3.13)
G304R	1 (1.8)	2002 (2.6)	1.0000	0.69	(0.09-4.95)
R202Q	2 (3.6)	2541 (3.3)	0.7067	1.09	(0.27-4.48)
E148Q	10 (17.9)	17561 (22.7)	0.5224	0.74	(0.37-1.47)
L110P	2 (3.6)	5354 (6.9)	0.4358	0.50	(0.12-2.05)
E84K	0 (0.0)	1253 (1.6)	1.0000	0.54	(0.03-8.71)

PMR: polymyalgia rheumatica, OR: odds ratio, CI: confidence interval. Allele frequencies are shown in parenthesis (%). Allele frequencies of PMR patients were compared with those of Japanese population controls by Fisher's exact test using 2×2 contingency tables under the allele model.
